# Analysis of Diabetes Mellitus-Related Amputations in the State of Espírito Santo, Brazil

**DOI:** 10.3390/medicina56060287

**Published:** 2020-06-11

**Authors:** Wendel Jose Teixeira Costa, Nilson Penha-Silva, Italla Maria Pinheiro Bezerra, Ismar Paulo dos Santos, José Lucas Souza Ramos, Jonathan Mendes de Castro, Júlio Eduardo Gomes Pereira, Alan Patrício da Silva, Adilson Monteiro, Luiz Carlos de Abreu

**Affiliations:** 1 Programa de Mestrado em Políticas Públicas e Desenvolvimento Local, Escola Superior de Ciências da Santa Casa de Misericórdia (EMESCAM), Vitória/Espírito Santo 29045-402, Brasil; enfermeirowendel@hotmail.com (W.J.T.C.); ismarps97@gmail.com (I.P.d.S.); lucas.enf15@gmail.com (J.L.S.R.); jonathanmendes1995@hotmail.com (J.M.d.C.); luiz.abreu@fmabc.br (L.C.d.A.); 2 Programa de Pós-graduação em Ciências Médicas. Faculdade de Medicina, Universidade de São Paulo. São Paulo 01246-000, Brasil; julioeduardo.pereira@gmail.com (J.E.G.P.); alanpatricio.fmabc@gmail.com (A.P.d.S.); adilmontero@hotmail.com (A.M.); 3 Universidade Federal de Uberlândia. Instituto de Biotecnologia. Uberlândia, Minas Gerais 38400-000, Brasil; 4 Programa de Mestrado em Ciências da Saúde da Amazônia, Bolsista CAPES Brasil, Universidade Federal do Acre (UFAC), Rio Branco/Acre 69915-900, Brasil; 5 Master in Public Health, Graduate Entry Medical School, University of Limerick, V94 T9PX Limerick, Ireland

**Keywords:** diabetes mellitus, amputation, health promotion

## Abstract

*Background and objectives:* Diabetes mellitus (DM) stands out among the most important public health problems worldwide since it represents a high burden on health systems and is associated with higher hospitalization rates, and a higher incidence of cardiovascular diseases. Amputations are among the most common complications, leading to disability and increasing care costs. This research aims to analyze the prevalence of DM-related amputations, comorbidities and associated risk factors in the diabetic population residing in the State of Espírito Santo, Brazil. *Materials and Methods:* This is a quantitative, exploratory, cross-sectional study with a time series design and the use of secondary data registered and followed by the system of Registration and Monitoring of Hypertension and Diabetes—SisHiperdia. *Results:* The sample consisted of 64,196 diabetic patients, out of them, 3.9% had type 1 DM, 10.9% with type 2 DM, and 85.2% with DM coexisting with hypertension. Most were female (66.6%), aged 40 to 59 years (45.6%), and 60 years and older (45.2%). The prevalence of DM-related amputations in the analyzed sample was 1.2% in type 1 DM, 1.5% in type 2 DM, and 2.2% in concomitant DM and hypertension. Higher amputation rates were observed in males in the age group above 60 years in type 1 DM and type 2 DM and were slightly higher in the age groups up to 29 years in DM with hypertension. A higher prevalence of amputation was related to smoking, physical inactivity, acute myocardial infarction (AMI), stroke, chronic kidney disease (CKD), and diabetic foot (DF) in all types of DM. *Conclusions:* The present study showed a significant prevalence of DM-related amputations. An increased prevalence was evidenced when correlated with smoking, physical inactivity, AMI, stroke, CKD, and DF with significant statistical associations, except for a sedentary lifestyle in type 1 DM.

## 1. Introduction

Diabetes mellitus (DM) stands out among the most important public health problems. It is among the top ten causes of death worldwide, reaching approximately four million deaths of people aged 20 to 79 years in 2017. In the same year, the prevalence of DM was estimated at 8.8% of the world population aged 20 to 79 years, totaling around 425 million people, with a trend for the year 2,045 of 9.9%, reaching 628.6 million citizens worldwide. Together with cardiovascular disease, cancer, and respiratory disease, it represents over 80% of all premature deaths from chronic noncommunicable diseases (NCDs) [1].

DM imposes high burden on health systems and is associated with higher hospitalization rates, higher utilization of health services, as well as a higher incidence of cardiovascular and cerebrovascular diseases, blindness, renal insufficiency, and non-traumatic lower-limb amputations. Diabetic foot ulcers and associated amputations are among the most common complications, leading to disability and increasing care costs [2,3]. Foot ulcers affect 4% to 10% of people with DM. About 40% to 60% of non-traumatic lower-limb amputations occur in these patients, and 85% of them are preceded by foot ulcers [4,5].

A study carried out with 21 countries from the Organization for Economic Cooperation and Development (OECD) showed an average rate of major amputations equal to 7.5 per 100,000 in the general population and 128.3 per 100,000 in the diabetic population. For minor amputations, the standardized rate for age and sex was 11.1 per 100,000 in the general population and 184.3 per 100,000 in the diabetic population [6].

The frequency of amputation is 10 to 20 times higher in the diabetic population compared to non-diabetics [7]. Worldwide, one million individuals with DM suffer lower limb amputation annually, accounting for about three amputations per minute [8].

In Brazil, between 2011 and 2016, 102,056 amputation surgeries were performed by the National Health System (SUS—Sistema Único de Saúde); out of them, 70% were diabetic individuals, with 94% of these being lower-limb amputations [9]. Estimates indicate that for a population of 7.12 million diabetics, 484,500 would develop foot ulcers, 169,600 would be admitted to hospitals and 80,900 would suffer some kind of amputation, of them, 21,700 would die as a result [10].

A study on lower-limb amputations in the metropolitan region of Rio de Janeiro, Brazil, showed an incidence of 13.9 per 100,000 inhabitants for the general population, and 180.6 per 100,000inhabitants for the diabetic population [11]. 

Individuals with DM suffer lower limb amputations for many reasons, such as age, disease duration, prolonged hyperglycemia, dyslipidemia, smoking, excessive use of alcohol, infections, peripheral neuropathy, ulcers, and inadequate metabolic control of the disease [12,13].

Peripheral arterial disease (PAD) and lower extremity diabetic peripheral neuropathy (DPN) are associated with DM-related amputations [8]. The survival rate for DM patients in the three years following an amputation is about 50% [12].

DPN can lead to loss of protective sensitivity, double the likelihood of foot ulcer development, and triple the risk of lower extremity amputation [14,15,16,17]. 

From this perspective, it has become evident that there is a need for commitment by government leaders and healthcare managers to create mechanisms that minimize the onset, as well as the aggravation of DM [18].

These efforts should be directed at both the reorganization of care for people with DM and the provision of necessary inputs for disease monitoring, with the aim of reducing the costs of early non-detection and complications related to lack of metabolic control [19].

Multi-professional care for people with DM and diabetic foot (DF) should be considered in public health policies, since better results have been achieved when the approach is given by multi-professional teams, sharing knowledge and expertise, reducing DM amputation rates [20].

The collection of data regarding complications of DM, the identification of the epidemiological profile, and the prevalence of amputations, as well as their correlations, are of great value for the elaboration, implementation, and evaluation of the results of public policies and health promotion programs. For these reasons, the present study aims to analyze the prevalence of DM-related amputations, comorbidities, and related risk factors in the diabetic population living in the state of Espírito Santo, Brazil.

## 2. Materials and Methods

### 2.1. Study Design

This is a descriptive cross-sectional study [21], incorporating a time series design and the use of secondary data on the morbidity of individuals with DM who underwent DM-related amputation, residing in the state of Espírito Santo, Brazil, registered and followed by the System of Registration and Follow-up of Hypertension and Diabetes—SisHiperdia.

### 2.2. Study Location and Period

Data were collected from the place of residence of individuals registered in the system. The unit of analysis selected was the State of Espírito Santo, Brazil. The data corresponded to the period between 2003 and 2012.

The period analyzed is justified since SisHiperdia was discontinued at the beginning of 2013, with no more recent data available. Unfortunately, there is no other information system in the country with similar data. However, the data used in this survey are complete, and reliably represents the profile of the population analyzed. This information platform contains important indicators of the results of public health policies implemented by the Brazilian government for monitoring hypertension and DM which had a significant impact on the current population residing in the State of Espírito Santo. 

The selection of this database is due to the representativeness and completeness of its information, making it appropriate to measure the magnitude of the problem in question and the possibility of directing the public power to the construction of effective strategies for monitoring diabetes and its complications.

### 2.3. Study Population and Eligibility Criteria

All individuals with type I DM, type II DM, and DM with concomitant arterial hypertension who underwent amputation due to DM, were a resident in the state of Espírito Santo, and were registered and followed by the SisHiperdia in the period of 2003 to 2012, were included.

No individuals were excluded from the sample. Individuals who reported as diabetic with concomitant arterial hypertension in SisHiperdia were not stratified by the type of diabetes (type I DM or type II DM).

The database includes amputations related to Diabetes but does not differentiate them from major and minor amputations, hence it is not possible to analyze these data separately. However, this fact did not compromise the objectives of this research.

### 2.4. Data Collection

Data were extracted from the SisHiperdia, provided by the Department of Informatics of the National Health System—DATASUS [22]. Two independent researchers organized the data in a Microsoft^®^ Excel version 15.0 spreadsheet. Then, an exploratory analysis was performed to recognize the variables and correct possible errors or inconsistencies in data entry. Once the necessary corrections were made, the data were organized and analyzed by applying descriptive statistics.

SisHiperdia is a health program monitoring system established by the Ministry of Health in 2002, as part of the Reorganization Plan for Arterial Hypertension and Diabetes Mellitus Care, consolidating itself as a platform for registration and monitoring of patients with hypertension and diabetes mellitus, assisted by the team of Primary Care of the SUS. This database generates information on the epidemiological profile of the population with hypertension and diabetes mellitus, which is available for professionals and managers of municipal and state departments and the Ministry of Health. This information is publicly accessible with the exception of patient identification [23].

This system presents several options for the use of its data and makes it possible to investigate whether the population at risk is adequately assisted, to know the demographic and epidemiological profile of the affected community, to verify the prevalence of risk factors, concomitant diseases, and complications, monitor the clinical quality of care provided and determine the real parameters of the continuous supply of medicines. Moreover, it enables social control through information to analyze access, coverage and quality of care [23,24].

Data collected by municipalities are transferred via the internet, consolidated by the Ministry of Health, and made available monthly for public access on the DATASUS website, allowing access to reports with operational, managerial, and epidemiological indicators predefined by the National Coordination of the Hypertension Program and Diabetes (CNPHD). Users can also access key system database information, calculate indicators and produce charts and maps from their own fast tabs using the free Tabnet and Tabwin applications available from DATASUS [23,24].

### 2.5. Data Analysis

The prevalence of DM amputation, stratified by type I DM, type II DM, and DM with concomitant arterial hypertension, by gender and age group, by comorbidities and associated risk factors (smoking, physical inactivity, overweight, acute myocardial infarction (AMI), stroke, chronic kidney disease (CKD), and DF) were calculated and expressed as a percentage using the direct method.

Prevalence ratios (PR) were calculated, and associations between amputations with risk factors and comorbidities reported in the system were analyzed. The dependent variable (y) was DM amputation, while the independent variables (x) were gender, age, smoking, physical inactivity, overweight, AMI, stroke, CDK, and DF. Associations were analyzed by Pearson’s chi-squared test and the Yates correction, using the Stata®14.0 statistical program and considering a 95% confidence level.

### 2.6. Ethical and Legal Aspects of the Research

This study involved only the description and analysis of secondary data collected from the SisHiperdia, informed by DATASUS. All of these sources of information are in the public domain. No individually identifiable information was obtained for this study.

## 3. Results

The sample consisted of a total of 64,196 diabetic individuals, residents in the state of Espírito Santo. Table 1 illustrates the characterization of the sample by type of DM, gender, and age group.

### Prevalence of DM-Related Amputations

The prevalence rates of DM-related amputations stratified by type of DM, gender, age group, and associated risk factors and comorbidities are shown in Table 2, Table 3 and Table 4.

The prevalence of DM-related amputations in the analyzed sample was 1.2% (*n* = 29) in type 1 DM, 1.5% (*n* = 104) in type 2 DM, and 2.2% (*n* = 1224) in DM with hypertension. Higher amputation rates were observed in males over the age of 60 years in type 1 DM and type 2 DM and were slightly higher in the age group up to 29 years in DM with hypertension. 

The correlation between amputations and associated risk factors and comorbidities showed a higher prevalence of smoking, sedentary lifestyle, AMI, stroke CKD, and DF in all types of DM, with a statistically significant association, except for sedentary lifestyle in type 1 DM. The prevalence of amputations was lower in the overweight population with type 1 DM, type 2 DM, and coexistent hypertension and DM, showing a statistically significant association in DM with hypertension.

## 4. Discussion

### DM-Related Amputations

The prevalence of DM-related amputations in the analyzed population was higher in type 2 DM, *n* = 104, 1.5% (95% confidence interval (CI) 1.2–1.8), and even higher in the coexistence of DM and hypertension, *n* = 1224, 2.2% (95% CI 2.1–2.4).

Our results are similar to those found in Canada, where the estimated prevalence of type 2 DM amputations was 1.4% [25]. In England, the age- and sex-adjusted incidence of minor amputations was 1.2 per 100,000 inhabitants in type 1 DM and 4.1 per 100,000 inhabitants in type 2 DM [26]. 

In Italy, a study reported a crude lower limb amputation rate of 20.4 per 100,000 inhabitants, 247.2 for 100,000 people with DM, and 8.6 for those without DM, demonstrating an increased risk of amputations in people with DM [27]. 

In type 1 diabetics, the lower prevalence of amputations may be associated with better glycemic control, related to insulin use, slowing the progression of the pathophysiological pathway of microvascular complications, and associated with improved skin microcirculation and lower incidence of ischemic foot ulcers [28]. The higher prevalence among people with DM and hypertension may be associated with the fact that hypertension significantly contributes to microvascular and macrovascular changes in DM, responsible for the pathophysiological changes in these cases [29].

In Minas Gerais, diabetic patients undergoing major amputation in a hospital in Belo Horizonte had a high prevalence of hypertension (54.7%) and an increased risk of a major amputation related to hypertension [30]. A cohort study conducted at a university hospital in the state of Pará, Brazil, with 711 individuals, showed an association of hypertension with the severity of PAD and a consequent increased risk for amputation [31]. 

A higher prevalence of amputations was found in males in all types of DM, with a statistically significant association. These findings converge with the Eurodiale study, which also demonstrated a higher risk and higher prevalence of male amputations [5,32]. 

A study conducted in the state of São Paulo also found a higher prevalence in males (57.5% to 61.3%), but without a statistical correlation with increased risk [33]. In Santa Catarina, the data are similar to those found in this study, with 64.9% of DM-related amputations occurring in males [34].

The higher prevalence of injuries and amputations in males may be associated with the fact that men are more reluctant to seek health care and follow preventive recommendations, and tend to be more neglectful of their own health [35,36].

However, recent research has shown that women with a history of DF have an equal or slightly higher risk for cardiovascular events, CKD, and mortality compared to men, with an emphasis on the increased risk of ischemic stroke [37]. Inequalities in treatment related to gender or socioeconomic conditions, hormonal factors, genetic predisposition linked to sexual dimorphism, alone or together, may be associated with an increased risk of cardiovascular complications in women [37,38,39].

In the age group above 60 years, the prevalence of amputations in the population studied was higher among individuals with type 1 DM; however, in the population with concomitant DM and hypertension, a slightly higher prevalence was observed in the age group up to 29 years. A statistical analysis showed a significant association only in type 2 DM.

In Belo Horizonte, an increase in the prevalence of amputations and mortality was shown in diabetic patients with advancing age (between 31 and 60 years *n* = 40 (15.4%), between 61 and 80 years *n* = 79 (22.7%) and older than 80 years *n* = 16 (38.1%), as well as an association with a higher risk of amputation in advanced age (odds ratio (OR) 1.05, *p* < 0.001), which corroborates our findings [30].

In Germany, researchers have shown a high prevalence and higher risk of PAD among type 1 diabetic elderly patients with a mean age of 62.6 years and in type 2 DM with a mean age of 71.3 years (*p* < 0.0001), probably related to a longer disease course [40].

Hypertension has been associated with accelerated progression of microvascular and macrovascular complications in the young adult population with associated DM and hypertension [29,41,42]. However, it was not possible to analyze, based on the information available in the researched database, whether the increased prevalence of amputations in young diabetic adults was directly related to hypertension.

A high prevalence of DM-related amputation was observed when correlated with smoking, physical inactivity, AMI, stroke, CKD, and DF in type 1 DM, type 2 DM, and concomitant hypertension DM, with a significant statistical association, with an excess of a sedentary lifestyle in DM type 1.

The prevalence of amputations was lower in the overweight population with type 1 DM, type 2 DM, and concomitant hypertension and DM, presenting a statistically significant association only in the diabetic population with hypertension.

Converging with our findings, a cohort study in a Saudi Arabia population of 62,681 diabetics over 25 years of age reported that smoking, CKD, coronary heart disease, cerebrovascular disease, and obesity were associated with a higher risk of foot complications in people with DM, including gangrene and amputations [43].

In Taiwan, a sample of 1,588 persons with type 2 DM undergoing non-traumatic amputation showed a higher prevalence of amputations in patients with a history of coronary artery disease and stroke [44].

The results of this study also converge with those found in Ghana, where the average incidence of foot problems (including amputations) was 8.39% in 7,383 people with diabetes with increased risk in males (OR = 2.51), in hypertension (OR = 1.14), nephropathy (OR = 2.15), and previously diagnosed DF (OR = 3.24), all with a statistically significant correlation. However, the increased risk for body mass index (BMI) increased by 5 kg/m² (OR = 3.2), which differs from the results found in the diabetic population with coexisting hypertension [45].

In Brazil, the Brazupa study evaluated 1,455 individuals with diabetes, and also showed a higher prevalence of amputations in males (70.9%; *p*< 0.001) in the elderly (mean age 60.5 years; *p* < 0.001), in type DM 2 (95.8%; *p* = 0.017), CKD (33.3%; *p* = 0.001), heart disease (36.4%; *p* = 0.023), previous DF changes (96.7%; *p* < 0.001), and among smokers (26.1% *p* = 0.52); however, the last one did not have a statistically significant association [46].

Smoking has been associated as a triggering or aggravating factor for type 2 DM, due to its effects on cortisol concentrations, inflammatory markers, oxidative stress, insulin resistance, as well as an increase in fasting glucose, contributing to vascular and neuropathic complications [47,48].

Sedentary lifestyle and smoking were associated with increased cardiovascular risk [49]. A sedentary lifestyle is related to increased insulin resistance and glycemic rates with pathophysiological repercussions on diabetic neuropathy and on micro and macrovascular changes [50].

Diabetics with mild to moderate CKD are more at risk for DF and amputations [51], and greater morbidity and mortality related to cardiovascular and cerebrovascular diseases were also associated, which justifies the findings of this research [52].

Vascular disease is related to a series of pathophysiological mechanisms that affect the structure and function of sago vessels, leading to arterial failure, the main risk factors being advanced age, DM, hypertension, hyperlipidemia, and smoking [53].

A high prevalence of vascular diseases has been shown in people with CKD, and even higher in albuminuria [54]. When CKD is associated with DM, a higher prevalence of critical ischemia and lower-limb amputations was observed [55]. Studies have pointed out that PAD and arterial stiffness are associated with worsening the progression of kidney disease and cardiovascular disease [56,57].

The frequency of amputations among people with diabetes is 10 to 20 times higher compared to the general population [7]. Each year, about one million individuals with diabetes undergo lower-limb amputation worldwide, accounting for about three amputations per minute [8]. DF is the leading cause of non-traumatic lower-limb amputations worldwide. In China, an amputation rate of 19.03% has been demonstrated in individuals with DM, which is 15 times higher than in those without diabetes [58]. 

Poor glycemic control, smoking, presence of hyperkeratosis, callus, and foot deformities, as well as visual impairment, CKD, previous history of ulcer and amputation, NP and PAD are the main risk factors for DF ulcers and amputations [59].

PAD is directly related to amputations, and its risk factors include DM, hypertension, dyslipidemia, smoking, and obesity. A recent meta-analysis showed an increased risk of PAD in patients with mild to moderate CKD [51]. Increased morbidity and mortality related to coronary and cerebrovascular disease were also found [52].

Often, diabetic people are affected by PAD and NP at the same time; however, the incidence of purely ischemic ulcers is not uncommon and is associated with local trauma [60]. The reduction in blood flow in the skin can make the vascular network more sensitive to occlusion during periods of high biomechanical pressure on the skin, contributing to the appearance of lesions and consequent amputation [61].

Foot ulcers and amputations are frequent and represent the main causes of morbidity and mortality in people with DM. Early detection and treatment of patients with DM and feet at risk for ulcers and amputations may delay or prevent adverse outcomes [59].

The high prevalence rates of DF-associated amputations, as evidenced by the present study, are justified by the direct association of DF injuries as precursors of amputations. DF is responsible for about 40% to 60% of non-traumatic lower-limb amputations in the world, with 85% of these amputations preceded by foot ulcers [4,5]. 

Lower limb amputations are a sentinel event because the risk is influenced by the control of various factors (glycemic control, blood pressure control, smoking, etc.) and depends on the ability of health systems to track the risk, stratify it, and treat patients with ulcers and high-risk feet [2].

The prevalence of DF and the consequent amputation is directly related to the organization of health services and the investment made in the treatment of DM, which varies in each country, and is, therefore, more prevalent in poorer countries [62].

Late diagnosis is associated with poor health system performance, poor awareness of the disease, including among health professionals, and the silent progression of the disease, favoring the development of its complications. Governments and public health systems in many countries are still unaware of the current relevance of DM and their complications [18,63].

Given the factors that directly affect the development of DF, we realize the need that individuals with diabetes should have to maintain self-care and also the importance of the multidisciplinary team to ensure the quality of life of these patients, advising on the disease and its risks [64].

Another research demonstrated the importance of a new look at the work processes, aiming at the individual's health instead of just looking at the disease [64]. In addition, it is essential to carry out educational methods with patients to improve health promotion and education practices, these practices are fundamental for the delivery of services and assistance to individuals with chronic diseases, ensuring the change in the care model [65,66].

Upon analyzing our findings, we realized the importance of implementing public health policies to monitor the control of risks related to DM. Ensuring comprehensive care, self-care education, and routine examination of the feet of people with diabetes, especially at the primary level of health care, can reduce these rates and consequently improve the health and quality of life of people with DM.

Data collection regarding the complications of DM is an instrument capable of directing the attention of the public authorities and enables the planning of actions, directed to the prevention and control of comorbidities and risk factors. 

Comparative data on DM-related amputations by type of DM are relatively scarce, and in the databases searched, no Brazilian study evaluating and comparing the prevalence of amputations by type of DM in a population similar to this study was found, corroborating the relevance of this research.

The results show that the prevalence of amputations related to DM in the state of Espírito Santo is still high, requiring constant monitoring and periodic review of strategies to implement public policies for managing this disease, and likewise, the development of new studies that separately analyze major and minor amputations, and identify the main difficulties encountered by the government, health professionals, patients and family members in coping with DM and its complications is required.

Studies that analyze major and minor amputations separately are an innovative trend in the scientific field and are configured as a more effective strategy for the evaluation of health policy outcome indicators.

## 5. Conclusion

It was found a significant prevalence of DM-related amputations in the analyzed population, with a higher prevalence after 60 years of age and in males. An increased prevalence was correlated with smoking, physical inactivity, AMI, stroke, CKD, and DF, with statistically significant associations, with the exception of a sedentary lifestyle in type 1 DM. The prevalence of amputations was lower in the overweight population, indicating a statistically significant association only in people who live with concomitant diabetes and hypertension.

## Figures and Tables

**Table 1:** The total number of diabetic individuals registered and followed by the SisHiperdia in Espírito Santo from 2003 to 2012, stratified by type of Diabetes Mellitus (DM), gender, and age group.

Variables	Total	%	Type I DM	%	Type II DM	%	DM with Hypertension	%
Sample	64,196	100	2512	3.9	6995	10.9	54,689	85.2
Gender								
Male	21,416	33.4	1138	45.3	2875	41.1	17,403	31.8
Female	42,780	66.6	1374	54.7	4120	58.9	37,286	68.2
Age group								
Up to 19	696	1.1	391	15.6	99	1.4	206	0.4
20 to 39	5219	8.1	672	26.8	995	14.2	3552	6.5
40 to 59	29,282	45.6	922	36.7	3,767	53.9	24,593	45.0
60 of age or older	28,999	45.2	527	21.0	2,134	30.5	26,338	48.2

Source: SisHiperdia (Department of Informatics of the National Health System - DATASUS), 2019.

**Table 2:** Prevalence% of DM-related amputations in the type 1 DM population, stratified by gender, age group, smoking, physical inactivity, overweight, acute myocardial infarction (AMI), stroke, chronic kidney disease (CKD), and diabetic foot (DF) in the state of Espírito Santo, Brazil, from 2003 to 2012.

Variables	Yes *n* (P%) 95% CI	No *n* (P%) 95% CI	PR (95%CI)	“*p*”
Amputation	29 (1.2) 0.8–1.7	2483 (98.8) 98.3–99.2		
Gender				
Male	21 (1.8) 1.2–2.9	1117 (98.2) 97.1–98.8	3.17 (1.41–7.13)	0.0057
Female	8 (0.6) 0.3–1.2	1366 (99.4) 98.8–99.7		
Age group				
Up to 29	6 (0.8) 0.3–1.9	701 (99.2) 98.1–99.7	1.79 (0.62–5.12)	0.5505
30 to 59	15 (1.2) 0.7–2	1263 (98.8) 98.0–99.3	1.29 (0.55–3.03)	
60 and +	8 (1.5) 0.7–3.1	519 (98.5) 96.9–99.3		
Risk factors and comorbidities				
Smoking				
Yes	9 (2.6) 1.3–5	340 (97.4) 95–98.7	2.79 (1.28–6.08)	0.0158
No	20 (0.9) 0.6–1.5	2143 (99.1) 98.5–99.4		
Sedentary lifestyle				
Yes	14 (1.5) 0.8–2.6	926 (98.5) 97.4–99,2	1.56 (0.76–3.22)	0.3067
No	15 (1) 0.6–1.6	1557 (99) 98.4–99.4		
Overweight				
Yes	6 (1.1) 0.5–2.5	532 (98.9) 97.5–99.5	0.96 (0.39–2.34)	0.8953
No	23 (1.2) 0.8–1.8	1951 (98.8) 98.2–99.2		
AMI				
Yes	4 (6.1) 2–15.6	62 (93.9) 84.4–98	5.93 (2.12–16.56)	0.0014
No	25 (1) 0.7–1.5	2421 (99) 98.5–99.3		
Stroke				
Yes	3 (4.8) 1.3–14.4	59 (95.2) 85.6–98.7	4.56 (1.42–14.66)	0.0317
No	26 (1.1) 0.7–1.6	2424 (98.9) 98.4–99.3		
CKD				
Yes	8 (7.2) 3.4–14.1	103 (92.8) 85.9–96.6	8.24 (3.73–18.19)	<0.0001
No	21 (0.9) 0.6–1.4	2380 (99.1) 98.6–99.4		
DF				
Yes	17 (23) 14.3–34.5	57 (77) 65.5–85.7	46.67 (23.13–94.16)	<0.0001
No	12 (0.5) 0.3–0.9	2426 (99.5) 99.1–99.7		

*n*—number of individuals; P%—Percent Prevalence; CI—Confidence interval; PR—Prevalence ratio; “*p*”—*p* value by Pearson's chi-square test and Yates correction. Source: SisHiperdia (Department of Informatics of the National Health System - DATASUS), 2019.

**Table 3:** Prevalence % of DM-related amputations in the type 2 DM population, stratified by gender, age group, smoking, physical inactivity, overweight, acute myocardial infarction (AMI), stroke, chronic kidney disease (CKD), and diabetic foot (DF) in the state of Espírito Santo, Brazil, from 2003 to 2012.

Variables	Yes *n* (P%) 95% CI	No *n* (P%) 95% CI	PR (95% CI)	“*p*”
Amputation	104 (1.5) 1.2–1.8	6891 (98.5) 98.2–98.8		
Gender				
Male	66 (2.3) 1.8–2.9	2809 (97.7) 97.1–98.2	2.49 (1.67–3.7)	<0.0001
Female	38 (0.9) 0.7–1.3	4082 (99.1) 98.7–99.3		
Age group				
Up to 29	3 (0.9) 0.2–2.9	321 (99.1) 97.1–99.8	2.33 (0.73–7.44)	0.0084
30 to 59	55 (1.2) 0.9–1.6	4482 (98.8) 98.4–99.1	1.78 (1.21–2.62)	
60 and +	46 (2.2) 1.6–2.9	2088 (97.8) 97.1–98.4		
Risk factors and comorbidities				
Smoking				
Yes	26 (2.3) 1.5–3.4	1097 (97.7) 96.6–98.5	1.74 (1.12–2.7)	0.0178
No	78 (1.3) 1.1–1.7	5794 (98.7) 98.3–98.9		
Sedentary lifestyle				
Yes	64 (1.9) 1.5–2.4	3338 (98.1) 97.6–98.5	1.69 (1.14–2.5)	0.0107
No	40 (1.1) 0.8–1.5	3553 (98.9) 98.5–99.2		
Overweight				
Yes	33 (1.2) 0.8–1.7	2690 (98.8) 98.3–99.2	0.73 (0.48–1.1)	0.1570
No	71 (1.7) 1.3–2.1	4201 (98.3) 97.9–98.7		
AMI				
Yes	18 (11.6) 7.2–18	137 (88.4) 82–92.8	9.24 (5.7–14.96)	<0.0001
No	86 (1.3) 1–1.6	6754 (98.7) 98.4–99		
Stroke				
Yes	17 (11.2) 6.8-17.6	135 (88.8) 82.4-93.2	8.8 (5.37-14.42)	<0.0001
No	87 (1.3) 1-1.6	6,756 (98.7) 98.4-99		
CKD				
Yes	20 (6.8) 4.3–10.5	272 (93.2) 89.5–95.7	5.47 (3.4–8.77)	<0.0001
No	84 (1.3) 1–1.6	6619 (98.7) 98.4–99		
DF				
Yes	71 (31.1) 25.3–37.6	157 (68.9) 62.4–74.7	63.86 (43.18–94.44)	<0.0001
No	33 (0.5) 0.3–0.9	6734 (99.5) 99.1–99.7		

*n*—number of individuals; P%—Percent Prevalence; CI—Confidence interval; PR—Prevalence ratio; “*p*”—*p* value by Pearson's chi-square test and Yates correction. Source: SisHiperdia (Department of Informatics of the National Health System - DATASUS), 2019.

**Table 4:** Prevalence % of DM-related amputations in the population with DM and hypertension, stratified by gender, age group, smoking, physical inactivity, overweight, acute myocardial infarction (AMI), stroke, chronic kidney disease (CKD), and diabetic foot (DF) in the state of Espírito Santo, Brazil, from 2003 to 2012.

Variables	Yes *n* (P%) 95% CI	No *n* (P%) 95% CI	PR (95% CI)	“*p*”
Amputation	1224 (2.2) 2.1–2.4	53,465 (97.8) 97.6–97.9		
Gender				
Male	492 (2.8) 2.6-3.1	16,911 (97.2) 96.9–97.4	1.44 (1.29-1.61)	<0.0001
Female	732 (2) 1.8-2.1	36,554 (98) 97.9–98.2		
Age Group				
Up to 29	23 (2.5) 1.6–3.7	908 (97.5) 96.3–98.4		0.0540
30 to 59	572 (2.1) 1.9–2.3	26,848 (97.9) 97.7–98.1	1.18 (0.78–1.79)	
60 and +	629 (2.4) 2.2–2.6	25,709 (97.6) 97.4–97.8	1.03 (0.69–1.56)	
Risk factors and comorbidities				
Smoking				
Yes	392 (4.4) 4–4.9	8434 (95.6) 95.1–96	2.45 (2.18–2.75)	<0.0001
No	832 (1.8) 1.7–1.9	45,031 (98.2) 98.1–98.3		
Sedentary lifestyle				
Yes	686 (2.4) 2.3–2.6	27,464 (97.6) 97.4–97.7	1.20 (1.08–1.34)	0.0013
No	538 (2) 1.9–2.2	26,001 (98) 97.8–98.1		
Overweight				
Yes	565 (2) 1.9–2.2	27,271 (98) 97.8–98.1	0.83 (0.74–0.92)	0.0009
No	659 (2.5) 2.3–2.6	26,194 (97.5) 97.4–97.7		
AMI				
Yes	369 (6.8) 6.1–7.5	5,078 (93.2) 92.5–93.9	3.9 (3.46–4.39)	<0.0001
No	855 (1.7) 1.6–1.9	48,387 (98.3) 98.1–98.4		
Stroke				
Yes	362 (6.8) 6.2–7.5	4,951 (93.2) 92.5–93.8	3.9 (3.46–4.4)	<0.0001
No	862 (1.7) 1.6–1.9	48,514 (98.3) 98.1–98.4		
CKD				
Yes	396 (8.5) 7.7–9.4	4250 (91.5) 90.6–92.3	5.15 (4.59–5.78)	<0.0001
No	828 (1.7) 1.5–1.8	49,215 (98.3) 98.2–98.5		
DF				
Yes	726 (29.3) 27.5–31.1	1754 (70.7) 68.9–72.5	30.69 (27.58–34.15)	<0.0001
No	498 (1) 0.9–1	51,711 (99) 99–99.1		

n—number of individuals; P%—Percent Prevalence; CI—Confidence interval; PR—Prevalence ratio; “*p*”—*p* value by Pearson's chi-square test and Yates correction. Source: SisHiperdia (Department of Informatics of the National Health System - DATASUS), 2019
